# Is there more than the sewage plant? University freshmen’s conceptions of the urban water cycle

**DOI:** 10.1371/journal.pone.0200928

**Published:** 2018-07-19

**Authors:** Sarah Schmid, Franz X. Bogner

**Affiliations:** ZMNU (Centre of Math & Science Education), Department of Biology Education, University of Bayreuth, Bayreuth, Bavaria, Germany; Universidade de Coimbra, PORTUGAL

## Abstract

The concepts of 340 university freshmen concerning urban water cycles include various misconceptions (or intuitive conceptions) which severely contrast with correct scientific ones. Almost no student knew the correct urban water cycle in total, including cycle steps in the appropriate sequence: consumer (given), sewage-plant, nature and waterworks. Concepts mainly omit nature and waterworks, only the sewage plant is included in almost all concepts. This reflects an exaggeration of the importance of the cycle-step sewage plant relative to the other steps, when the topic is taught in school. Students acquired knowledge from two main sources: School and media. Most students are aware of the origin of drinking water, although several concepts reflect a pipe-to-pipe system, where wastewater is cleared in sewage plants and brought back to consumers, skipping the roles of nature and waterworks. Everyday matters with an important impact on our life-standards, like the urban water cycle, need specific attention during school time. Currently, only primary school syllabi include the issue of urban water cycles in Germany. More effort is needed to explain wastewater and drinking water issues in order to correct misconceptions.

## Introduction

### Misconceptions

Our concepts of things are often on the one hand based on personal explanations intuitively derived from everyday experiences, and on the other on scientifically correct explanations of experts such as teachers [1–3]. These origins can produce discrepancies, and the concepts we form while learning may differ from the scientifically correct explanation. Such discrepancies are labeled “alternative conceptions”, “intuitive conceptions” or “misconceptions” [4]. For practical reasons we prefer the term “misconception” in our paper. It is the most prominent term for concepts that deviate from the scientific view [4]. We are aware that in the past the term has been loaded with negative associations towards the learner [4] and that other terms have been established. However, we believe none of the other terms can give such a clear picture of a partially incorrect concept, without the need for long descriptions. When we use the term “misconception” in this paper, our interest is in the statements made. We do not rate the person behind a statement.

Even when people hold different concepts of the same thing, this need not lead us to favor one over the other, but either to use our concepts situation-dependently, or to integrate one concept into the other [1,5,6]. Scientifically incorrect conceptions are deeply rooted and not easy to overcome [1]. However, they can be corrected both in and out of school. Confronting students with common misconceptions may help them to recognize the difference between an incorrect and a scientifically correct explanation [7,8].

### Misconceptions about water

Access to clear drinking water is regarded as a human right [9]. In our modern world, the topic of water, especially the urban water cycle, should be of interest to all of us, as this water is the basis of all our tap water. Only if students understand how clear drinking water is provided, they may develop an understanding of their own responsibilities e.g., for protecting the environment by not pouring inappropriate substances into drains or littering rivers. Although in Germany tap water of drinking water quality is taken for granted by consumers, complex clearing procedures lie behind this service to guarantee high standards. Generally tap water in Germany is always of drinking water quality, as its high quality is tested on a regular basis [10], which makes it the best tested substance for oral consumption in Germany [11]. The quality standards for tap water are regulated through the “Drinking Water Ordinance 2001” [12]. 99% of Germany’s inhabitants have access to drinking water [11]. People trust this well-known quality of tap water and consume it regularly [13]. Raw-water is the water that is processed by waterworks. It mainly originates from ground water (68,1%; [10]) which is processed to drinking water in a few steps in waterworks before it is piped to households. A separate wastewater pipe system collects the “used” water and delivers it to sewage plants. Several steps ensure that the wastewater is cleared to a point where it does no longer affect nature too negatively; however it not yet is of drinking water quality and should not be consumed. The sewage plant output is returned to nature, e.g., inserted into rivers. Swimming in surface water can be a health risk due to sewage plant output. The natural process of clearing through the surface waters’ assimilative capacity and filtration through seepage is needed to obtain new ground water which can supply the waterworks [11]. The urban water cycle therefore consists of the stations: consumer, sewage plant, nature and water work.

When alternative conceptions about water are analyzed, the most prominent topic is the natural water cycle [14] or its physical and chemical properties [15]. Few studies exist concerning the beliefs and understanding of students regarding the human-controlled water cycle [13,16,17]. With the present study, we want to deepen our knowledge of what is known by students. Only informed citizens can fully take part in political discussions and understand the meaning of e.g. suggested changes or warnings concerning their drinking water and wastewater. If even young adults who have attained the highest school level hold misconceptions, they must be deeply rooted. Knowledge of these conceptions would help teachers and outside school educators to adapt their expectancies of students pre-knowledge as well as to target these misconceptions.

Cleaning processes are expected by the public, but what do young adults know about it? What do students know about the origin of drinking water? What does the urban water cycle look like in their minds? Are they well informed, or do we need to teach them better? In addition to open questions regarding the processes of the urban water cycle, we included questions on students’ perceived knowledge level and asked where they have obtained their information, to evaluate whether the topic is sufficiently discussed in school or if, for example, outreach programs would be a better way to combat these misconceptions. A broader range of school studies would be helpful in solving the problem of the formation of misconceptions about the urban water cycle.

Our study had four objectives:

1. How do university freshmen explain the urban water cycle?
2. Which misconceptions exist about the urban water cycle?
3. What are the origins of their related knowledge?
4. How high are perceived knowledge levels of students regarding drinking water and wastewater, and are they satisfied with their own level of knowledge?

## Material and methods

The research and consent processes of our study followed the requirements of the ethics committee of the University of Bayreuth. All participants were informed about the research conducted and provided their oral consent. Participation was voluntary. Data privacy laws were respected as our data was recorded pseudo-anonymously. Participants had the chance to reject study participation at any time. Our sample consisted of 340 German university freshmen, aged 19.5 years (+/-1.88 SD). 53.3 percent were female. Only students in their first semester were selected to participate to ensure that students had not attended lectures giving relevant information e.g. regarding the components of the water cycle. The questionnaires were completed at the start of the semester. As university students are assumed to be high achievers because they have obtained a university entrance diploma, their intuitive (but scientifically incorrect) conceptions are of particular interest.

We used a paper-and-pencil-questionnaire with 5 open and 5 closed questions. Completion required 10–15 minutes. Data was collected anonymously. The questionnaire was approved by the ethics committee of the University of Bayreuth. Through pilot testing and expert group rating the wording of our applied questions was optimized before data collection. The expert group consisted of 4 members of our department with experience in the design of open-question questionnaires.

For statistical analysis, SPSS 21 was used. Missing data was excluded list-wise. All open questions were categorized by the first author by using qualitative content analysis following [18]. 29% of qualitative data was randomly selected and additionally interrated by a non-project related person (Table 1). This person rated the entire 29% of data. The same random sample was additionally intrarated by the first author after 7 months (Table 1). Cohens-Kappa coefficients between 0.60 and 0.75 indicate a good level of agreement between the raters [19].

**Table 1:** The 5 open and 5 closed questions. Additionally Cohen’s kappa scores for intra-and interrater reliability for open questions are given. Number of raters for intrarating:1, for interrating:2.

Question		Reply	Cohens Kappa	
			Intrarater reliability	Interrater reliability
**Q1**	Where did you acquire your knowledge about wastewater?	open	0.94	1
**Q2**	Where did you acquire your knowledge about drinking water?	open	0.96	1
**Q3**	Where does the water originate that is processed into drinking water for you?	open	0.94	0.96
**Q4**	Describe the route the water takes until it emerges from your tap again. Domestic drain, …	open	0.96	0.94
**Q5a**	In your opinion, is there a difference between sewage plant and waterworks? (yes/no)	tick		
**Q5b**	In your opinion, are there differences between waterworks and a sewage plant? Describe!	open	0.78	0.95
**Q6a**	I feel well informed about drinking water. (rating)	tick		
**Q6b**	I feel well informed about wastewater. (rating)	tick		
**Q6c**	I would like to have more information about drinking water. (rating)	tick		
**Q6d**	I would like to have more information about wastewater. (rating)	tick		

Responses were used as the basis for inductive categorization (Table 2). This means that all answers were read and categories were formed while reading. The anchor example gives a definition of the category, while the category can be regarded as a headline. Statements were then assigned to categories. Answers containing more than one statement were assigned to that number of categories. For example, the answer “common knowledge” would be counted in one category, named “common knowledge”; the answer “everyday knowledge, guided tour in sewage plant, primary school” would lead to counts in three categories, named “common knowledge”, “guided tour in w.w. or s.p.” and “school”. Each statement was only counted once, even if one of the raters thought that it fitted more than one category; thus leading to the same number of statements and counts in the category-grid.

**Table 2:** Example of the category scheme for question Q1: Where do you have your knowledge about wastewater from?.

		categories								
		1: school	2: media	3: social contacts	4: common knowledge	5: guided tour in w.w. or s.p.	6: own experiences	7: other	8: I have no knowledge	9. no answer
**ID**	anchor example	School, primary, secondary, teacher, school lesson	TV, newspaper, internet, book, radio, documentaries, news	family, parents, friends, colleagues	every day knowledge, common knowledge	g.t. in waterworks, g.t. in sewage plant, g.t. without specification	volunteer work, own interest	not fitting other categories	I do not know; I have no knowledge about this.	No answer given; blank field
**001**	common knowledge				1					
**003**	everyday knowledge, guided tour in sewage plant, primary school	1			1	1				
**004**	school	1								
**…**	…	….	…	…	…	…	…	…	…	…

## Results

**Fig 1:**
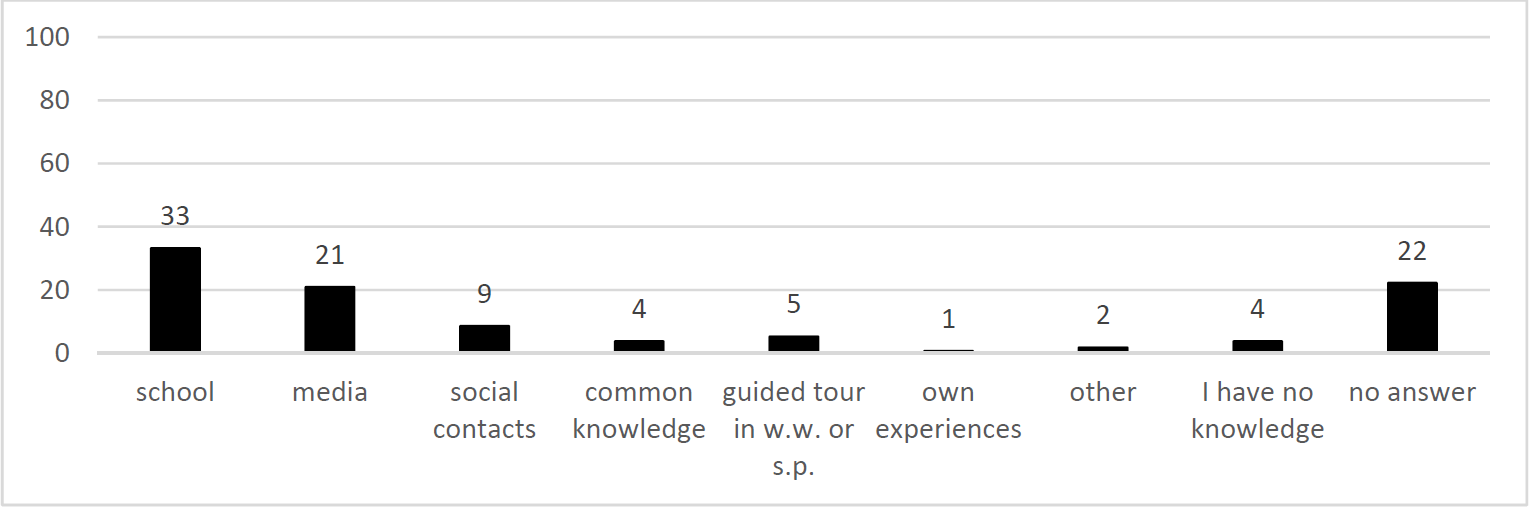
Statements included in responses to the question Q1: “Where did you acquire your knowledge about wastewater from?”. N_Students_ = 340, sum of statements = 100% (455 statements), y-axis = percentile amount of statements per category.

Question Q1 “Where did you acquire your knowledge about wastewater?” is illustrated in Fig 1. Most statements were pooled into the category “school” (33%), followed by “media” (21%), “no answer” (22%), “social contacts” (9%), “visits to sewage plants or waterworks” (5%). 4% of answers were count in “I have no knowledge”.

Had the category “school” not been grouped and instead subcategories used, 10% of all answers would include “explicitly elementary school”, 24% would only contain “school” with no further specification and less than 1% “secondary school”.

**Fig 2:**
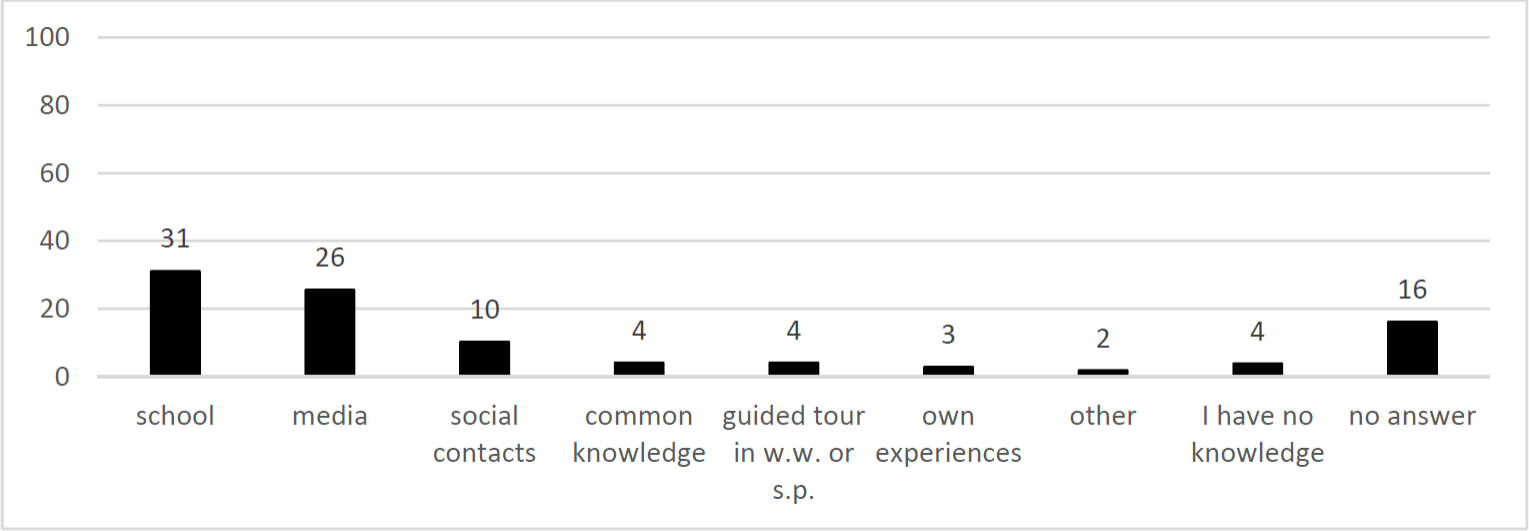
Statements included in responses to the question Q2: “Where did you acquire your knowledge about drinking water from?”. N_Students_ = 340, sum of statements = 100% (489 statements), y-axis = percentile amount of statements per category.

The answers of question Q2 “Where did you acquire your knowledge about drinking water?”, are illustrated in Fig 2 and contained the following categories the most: school (31%), media (26%), no answer (16%) and social contacts (10%). A further subcategorization of “school” allocated 10% of all answers explicitly to “elementary school”, 24% to “school” with no further specification and 1% to “secondary school”. (That the pooled category adds up to 35% is due to double statements of participants. They may have listed “school” and “elementary school” which would be counted only once for the main category “school”, but if subcategories were used instead they would be counted once in the category “school with no further specification” and once in “elementary school”).

**Fig 3:**
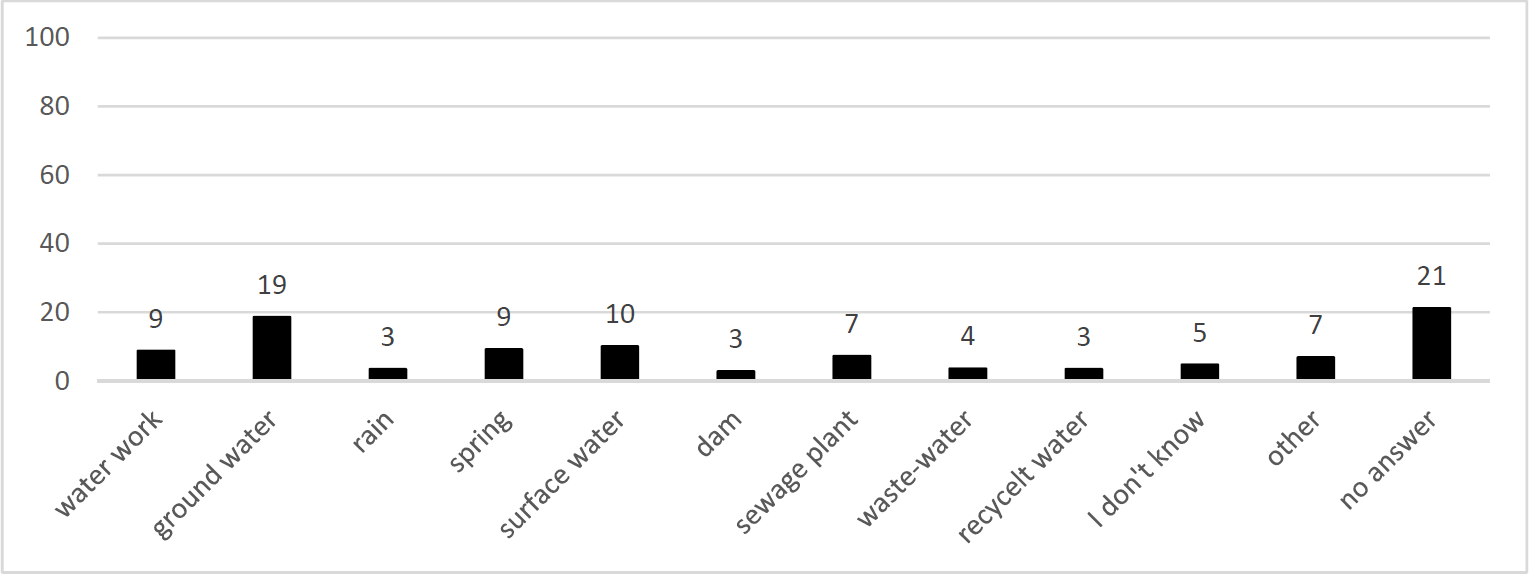
Statements included in responses to the question Q3: “Where does the water originate that is processed into drinking water for you?”. N_Students_ = 340, sum of statements = 100% (467 statements), y-axis = percentile amount of statements per category.

Question Q3 “Where does the water originate that is processed into drinking water for you?”, is illustrated in Fig 3. Most students did not answer at all (21% of statements). 19% of statements were pooled in the category “groundwater”, followed by “surface water” (10%) and “spring” (9%) as well as “waterworks” (9%). However, “sewage plant” was included in 7% of answers, “wastewater” 4%, “recycled water” 3% likewise. Therefore, the overall concept that drinking water can somehow be processed directly from wastewater is present in 14% of statements. The percentage of 14% was double-checked. It originates not only from the addition of the three categories “sewage plant”, “wastewater” and “recycled water”, which could include one person giving an answer including more than one of these categories. However, calculating a pooled category “some sort of wastewater” from the raw data, where an answer containing more than one of the categories (“sewage plant”, “wastewater”, “recycled water”) would lead to only one count, confirming the 14% for the pooled category “some sort of wastewater”.

**Fig 4:**
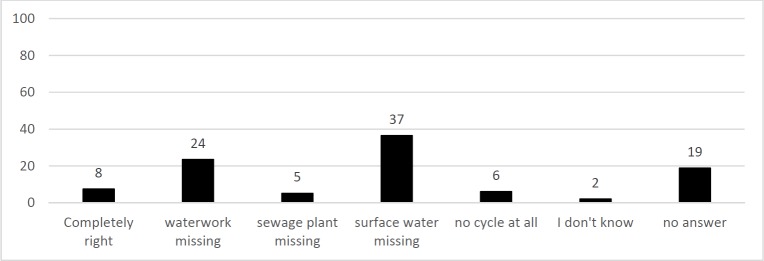
Statements included in responses to the question Q4: “Describe the route the water takes until it emerges from your tap again. **Domestic drain, …”.** N_Students_ = 340, sum of statements = 100% (424 statements), y-axis = percentile amount of statements per category.

Conceptions about the route water may take from drain to tap are illustrated in Fig 4. They were scientifically incorrect in 92% of all cases (and still in 72% of all cases, if one conservatively assumes that everyone who has given no answer would have known it correctly, but failed to write it down). Cycles were mostly described without surface water (37%) and/or waterworks (24%). The sewage plant was included in almost all concepts of the urban water cycle and was missing in only 5%. 19% of statements included no content.

**Fig 5:**
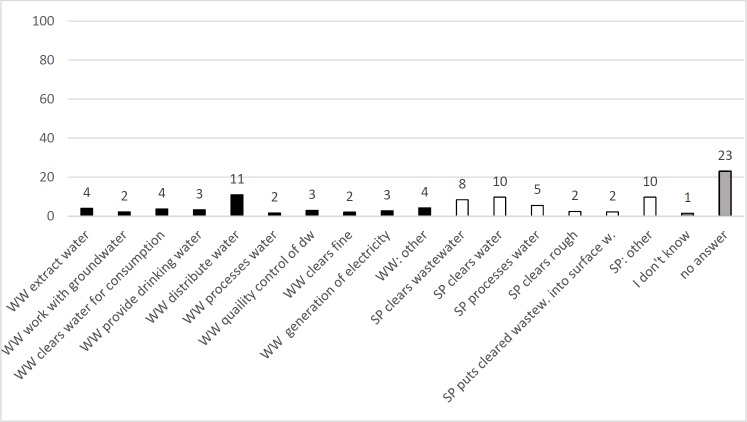
Statements included in responses to the question Q5b: “In your opinion, are there differences between waterworks and a sewage? **Describe!”.** N_Students_ = 340, sum of statements = 100% (547 statements), y-axis = percentile amount of statements per category.

For question 5a “In your opinion, is there a difference between sewage plant and waterworks?”, 74.56% of freshmen said, “yes” there is a difference between sewage plant and waterworks. 13.45% say “no”, there is no difference. 11.99% did not reply to this question. Results are illustrated in Fig 5.

Differences between sewage plant and waterworks were explained in a way that the main categories were “waterworks distribute water” (11%), “sewage plant clears water” (10%), “sewage plant clears wastewater” (8%) and “sewage plant processes water” (5%). Most students did not answer (23% of statements), 10% of statements (sewage plant) and 4% of statements (waterworks) belonged to aspects not fitting into categories, and 3% of statements included the misconception that waterworks generate electricity.

**Fig 6:**
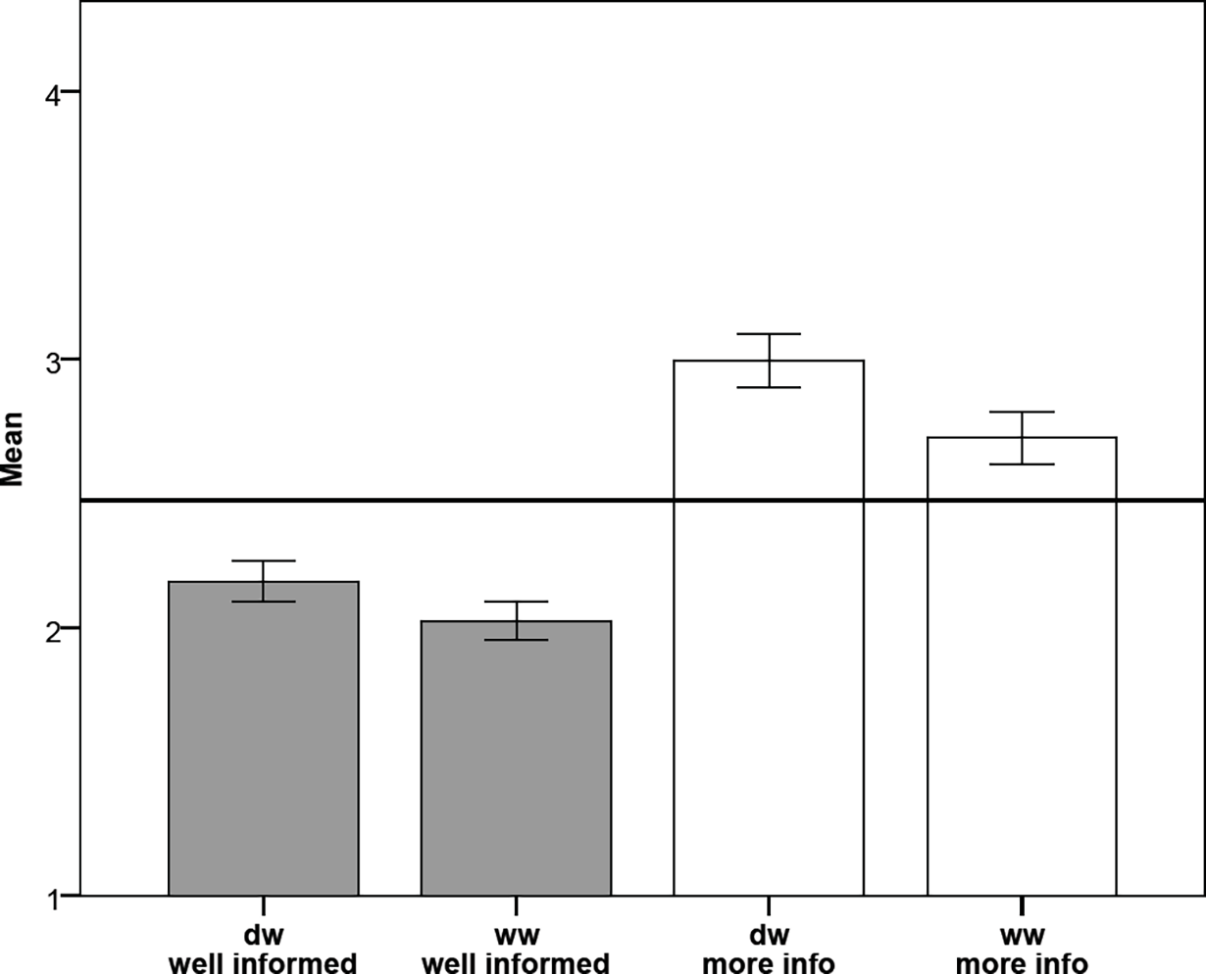
Mean values on four questions Q6a, b, c, d: about how well informed students feel and how much they would like to have more information about drinking water (dw) and waste water (ww). A Likert scale from 0 “not at all” to 4 “absolutely” was applied. Error bars CI = 95%.

Finally, students were asked to rate how well they feel informed about the topics drinking water (dw) and waste water (ww) and if they would like to have more information about them (Q6A,B,C,D; see Table 1; Fig 6). Possible ratings ran from 1 (refusal) to 4 (agreement).

The mean response implies that students felt rather ill-informed about drinking water and significantly worse informed about waste water (Wilcoxon rank-sum test: mean_dw_ = 2.17, SD = 0.70; mean_ww_ = 2.01, SD = 0.66; z = -4.31, p<0.00, r = -0.24; N = 331). Students want to know more about both topics, but information about drinking water was significantly more required than information about waste water (Wilcoxon rank-sum test: mean_dw_ = 2.99 SD = 0.92; mean_ww_ = 2.71 SD = 0.90; z = 7.42, p<0.00, r = -0.41; N = 331).

## Discussion

### The origins of existing knowledge

First of all, a large percentage of students replied that their knowledge about drinking water and also about waste water is based on information from school and media. Therefore school curricula seem to provide an effective mechanism to inform students about this topic. Using well-selected media in the classroom could additionally enhance understanding of the topic, as it was the second most frequent source of information. However, the media, too, can promote the formation of misconceptions [20,21] perhaps also in the case of the urban water cycle. Teachers need to select age-appropriate media that provides simplified and correct information. Teachers should be aware that they could hold misconceptions themselves [22] and should therefore double-check their assumptions at least against two high quality sources. Teachers holding misconceptions may not be able to detect these misconceptions in their students [21] and therefore cannot prevent their presence in students’ life. Using different sources of information might help teachers to recognize their own misconceptions: information from different sources contains different details, wording and views of the topic.

As each of the 16 states of Germany follows separate syllabi, comparing curricular standards about the urban water cycle is difficult. The urban water cycle or parts of it can be taught in biology, chemistry and geography classes. However, the topic is not compulsory in secondary school (Gymnasium syllabus in e.g. Baden-Württemberg, Bavaria, Berlin/Brandenburg [23–25]). On the other hand, the natural and urban water cycles are only compulsory topics in primary school throughout Germany, mainly in grade 4 (syllabus, grade 3+4 in e.g. Baden-Württemberg, Bavaria, Berlin/Brandenburg [26–28]). As many students replied that their knowledge about the urban water cycle explicitly originates from primary school, this underlines the importance of that source. Therefore, knowledge acquired in elementary school seems very important for life after school, which also means that this is the locus where misconceptions need to be overcome. A decade of a freshman’s life has passed with no or little new information on the topic. The formation of misconceptions therefore seems comprehensible.

### Misconceptions

The majority of students hold scientifically incorrect conceptions about the urban water cycle. School therefore failed to form literate citizens on the topic. This is in spite of all students having reached the highest possible school level, required for university entrance.

#### Drinking water made of wastewater

The misconception that drinking water is (in some way) made out of wastewater is included in 14% of statements of university freshmen (Q3). This finding is in line with [13], who reported this for younger students (age 16; 35%) and for freshmen (31%) and thus found this misconception twice as frequent as we did. In our sample, 9% of university freshmen named the waterworks as the source of their drinking water. However, most of our students named ‘ground water’ and ‘surface water’ as sources, probably because they misunderstood the question as asking where the waterworks acquire their water. The majority of students in our study therefore can be assumed to know that drinking water comes from natural resources or from waterworks. However, some students seem to hold the idea of a pipe to pipe system in Germany’s urban water cycle, where the sewage plant output is directly purified to drinking water quality. Our conclusion from Q3 is also present in answers of Q4, regarding the description of what the urban water cycle looks like. For example, student SEN-092 answered Q4: “Sewage plant, many filtrations, on to waterworks, control of minerals & bacteria-concentration, on further to consumer“. Or student SEN-055: “Sewage plant, waterworks, tap”; or even as short as student SEN-031: “canalization, sewage plant, tap”.

The formation of this misconception (that drinking water is derived from some sort of wastewater) might be explained by primary schools’ focus on the sewage plant when teaching the urban water cycle. The stages of the clearing process of the sewage plant are taught in detail. Very often outreach field trips to a sewage plant deepen the issue even further. If the water is dirty before and clean afterwards, why should it not be drinkable? We believe that underlining the fact that sewage-plant output-water is not drinkable and that there is a difference between “clean enough to be released into nature” and “clean enough for drinking” should help to overcome this misconception. Introducing cycles of the urban water cycle which do not “end” after the sewage plant and do not “start” at the consumer, could help too, as these two stages in the cycle—making drinking water dirty and making wastewater “clean”—are most likely already the two components most familiar to pupils. More time should be devoted to the processes in nature and waterworks in school lessons, so that they are perceived as equally important to students. [13] assume students and pupils mistake the word ‘sewage plant’ for ‘waterworks’. Also [17] reported the misconception of the sewage plant as the source of drinking water, discussing it as a form of over-generalization. However these explanations are unlikely to apply to our data, as students often use both terms in describing their ideas of the cycle. Additionally, contrary to [13], we found the term ‘waterworks’ used as the regular word for this stage in the cycle; descriptions like ‘water treatment plant’ did not occur. Not mentioning waterworks in the cycle therefore rather seems to be based on a non-scientific concept of the cycle than on difficulties to express oneself or missing knowledge of the term ‘waterworks’.

#### Basic separation of sewage plant (cleaning) and waterworks (transportation). No task left for nature

The question about differences between waterworks and sewage plant (Q5b) makes clear that the majority of freshmen address the task of water distribution to waterworks and the task of clearing water or specific wastewater to sewage plants. For example student DET-033 on Q5B: "The waterworks ‘only’ distribute water. The sewage plant cleans the water”. This basic separation could also explain misconceptions that neglect the cleaning processes of nature and waterworks. Only very few participants stated separation concerning the purification level (“SP clears rough”, “WW clears fine”). For example student DET-111 on Q5B: “Water from the waterworks needs to be cleaner“. So, if they regard the sewage plant as the station that makes dirty water clean, the waterworks may be seen as purely for transportation, so that there would be no role for nature: this would explain why it is so often not included. This likely over-generalization of “sewage plant cleans water” to “sewage plant cleans water to drinking water quality” has also been found in Ecuadorian students (age 16; [17]). The authors assume partial comprehension of the various stations of the urban water cycle as the source of the over-generalization.

The question about the urban water cycle (Q4) showed that only very few hold the scientifically correct concept of the urban water cycle consisting of the four major steps: consumer, sewage plant, nature, waterworks. Most cycles ignored the role of nature by not including surface water. Many cycles also missed waterworks. The component evident in nearly every conception was the sewage plant. The concept of direct recycling of wastewater through the sewage plant can be derived from these cycles. It seems that when school or public media inform about the urban water cycle, the greatest focus is on sewage plants. Furthermore, due to the visual change from dirty water to transparent water, students may mistakenly assume the transparent water to be of drinking water quality, as it superficially looks like tap-water. They may therefore ignore or forget the roles of surface water and waterworks in achieving drinking water quality. “Waterworks” seems to be a familiar term to many students, although many were able to describe their conception of the urban water cycle without it. Maybe this is because the actions of waterworks are harder to see (obtaining from nature, cleaning processes). Additionally, sewage plants can be smelled in everyday life, whereas waterworks cannot, which might reduce the knowledge of their existence or importance for the urban water cycle over time. Furthermore, waterworks are not prevalent in every city or region, as some regions receive water from far other regions, which is why many schools do not have the opportunity to take part in guided tours or experience waterworks. Using documentary video clips during class may compensate for this.

Perhaps, too, the neglect of this cycle step in primary school lessons explains the rare inclusion of nature in students’ concepts of the urban water cycle. Again, as the cleaning processes in surface water cannot be seen visually, and are even less easy to visit, this component is probably not included in guided tours. With the help of animations or experiments dealing with filtration, temperature or UV-light, the role of nature could be more emphasized.

#### Basic knowledge gaps but keen to know more

13.5% of respondents denied that sewage plants and waterworks differ (question 5a). This points to a lack of very basic knowledge and could help understanding why more complex topics like the urban water cycle are not well understood. Lack of knowledge is also illustrated in the question whether students feel well informed about drinking water and wastewater (Q6a, b). We conclude that freshmen are aware of their misconceptions or are unsure whether their suggestions are scientifically correct. Repeating this study in primary school would be interesting, as it could help to see whether pupils can overcome misconceptions if they are encouraged to ask questions to ascertain whether their mental image is in line with the scientific one. However, pupils’ conceptions could be scientifically correct immediately after learning about the urban water cycle, but deteriorate over time.

Additionally, freshmen wanted to know more about drinking water and about waste water (Q6c,d), with a higher demand for the first. This demand for information should be used by teachers whenever possible, since motivation and effort help to foster learning [29,30]. [16] reported that US-high school students held scientifically incorrect concepts of the water cycle, including human-engineered components. Only few high school students showed environmental science literacy. Likewise, we conclude that there is a risk that even high achievers are not fully able to participate in debates as environmentally literate citizens through school education.

Another reason for incomplete concepts might be that students learn and memorize the different cycle stations without connecting them to the next or previous cycle station. This conclusion was reached by [31] regarding concepts of the natural water cycle (students of 7^th^-9^th^ grade, junior high school, Israel). Our students apparently possessed basic knowledge of what a sewage plant does (cleaning water)–but they were mostly not capable of putting it into a larger context.

#### Implications for out of school education

Even high achievers are not immune to severe misconceptions and knowledge gaps: information provided by waterworks, sewage plants, politics or ecologically-oriented NGOs need to include very basic information to counteract those deficiencies. Messages such as “Be aware that conventional agriculture introduces nitrate into ground water” might not succeed in publicizing a potential health-threat, if no connection of ground water to the urban water cycle is added.

Outreach programs need to provide information on the complete urban water cycle before beginning a tour to avoid providing isolated information about the location to be visited. Helping visitors to understand which stage in the water circle e.g., a sewage plant represents may open their eyes to the importance of other circle steps. Including information about where water comes from, how dirt and detergents accompanying the feces get into their former drinking water and emphasizing that the water is not drinkable, even after the cleaning process of the sewage-plant, is important. Where water is released and where it is retrieved at waterworks, is important information to connect the main topic of how a sewage-plant functions with many other aspects of the urban water cycle. One barrier to generate this holistic view might be that the business of fresh-water-treatment and the business of wastewater-treatment are often provided by different entities.

#### Applications for in-school education

School education needs to focus on two major things. First, in primary school, attention must be paid to teaching all four cycle-components equally (waterworks, consumer, sewage plant, nature, waterworks…). Especially the role of nature in cleaning water needs to be stressed. Starting the topic of the urban water cycle with a broad view of the cycle function before looking closer at the different cycle steps, may help students to anchor knowledge about different cycle-components to certain parts in their concept of the water cycle, maybe preventing the tendency to skip stations in their mental image of the urban water cycle. In a second step, it may be helpful for students to understand the sequence of cycle-steps; and when confronted with incorrect cycles students should be asked to explain how they function or fail to function.

Furthermore, it could be of interest for teachers to find out students’ conceptions from time to time by asking them to describe certain processes before starting a topic. Misconceptions found in the literature can be a fruitful starting point for teachers. However, misconceptions in their class would be the best basis to confront students with misconceptions. Students would probably feel more motivated, as the ‘problem’ to solve would come from their own class, adding some importance to the topic. However, even extensive formal learning was found not to help overcoming misconceptions [4,32]. [33] found misconceptions regarding water pollution in Indian secondary school students (age ~16) and concluded that misconceptions should better be detected at an early stage, before they are linked to other information and become more resistant. Conceptual Change strategies were found to be fruitful instruments in overcoming misconceptions [32,34]. For first hand experiences, if possible, students should visit more than just a sewage plant. Visiting waterworks could hold much potential. Students would not only reactivate learned information at first hand, but the mere knowledge that they had visited two sites, each having another function in the urban water cycle, might help them to understand that drinking water does not directly come from a sewage plant. E.g., [35] showed cognitive knowledge to be enhanced through participation in a guided tour of waterworks.

#### How to overcome misconceptions

Students bring pre-knowledge from everyday life, gained from social contacts and modern media, to school. Incorrect reduction of details of information e.g., shown in public media, or taught in school may not only exacerbate the difficulty in understanding the information provided, but will also lead to the omission of certain details. An appropriate amount of didactic reduction (see e.g., [36] is crucial to providing information in an understandable manner to the learner. Too many details will result in cognitive overload and hinder learning [37]. Too much simplification will lead to gaps of knowledge and open the door to misconceptions. When details are not known, people need to connect isolated pieces of knowledge by thinking of plausible solutions by themselves. However, these solutions do not need to be scientifically correct, they merely need to satisfy the learner in connecting two parts of information [3,4]. New information is only integrated into existing concepts if the learner learns to recognize flaws in his current conception which would be corrected by accepting new information with more explanatory power [5]. This process, often called ‘Conceptual change’ [38], adds new information to existing concepts, extending the concept by connecting it to another viewpoint or topic, or giving it more depth by providing details–or replacing parts of it to make the concept more scientifically correct [5,39]. Provoking Conceptual Change has been shown to reduce misconceptions [40]. Additionally, the better the reasoning ability, the lower the number of misconceptions [41]. Promoting critical thinking skills and logical reasoning ability therefore seem to be one promising way of preparing students to build a mental image of a phenomenon closer to the scientific explanation.
